# Constitutive Activation of Skeletal Muscle mTORC1 Drives Pro‐Inflammatory Gene Expression in Skeletal Muscle of Aged Mice

**DOI:** 10.1111/acel.70805

**Published:** 2026-11-06

**Authors:** Erik D. Marchant, Hanna Kalenta, Jacquelyn May, Zhao Lai, Adam B. Salmon, Blake B. Rasmussen

**Affiliations:** ^1^ Department of Cellular and Integrative Physiology, Long School of Medicine University of Texas at San Antonio San Antonio Texas USA; ^2^ Barshop Institute for Longevity and Aging Studies University of Texas at San Antonio San Antonio Texas USA; ^3^ Department of Molecular Medicine, Long School of Medicine University of Texas at San Antonio San Antonio Texas USA; ^4^ Greehey Children's Cancer Research Institute University of Texas at San Antonio San Antonio Texas USA; ^5^ Geriatric Research Education and Clinical Center South Texas Veterans Healthcare System San Antonio Texas USA

**Keywords:** aging, geroscience, inflammaging, inflammation, mTOR, nutrient sensing

## Abstract

mTORC1 activity is increased in muscle with aging and is implicated as a potential driver of age‐related declines in muscle health. While pharmacological inhibition of mTORC1 improves lifespan and healthspan, it remains unclear whether chronic activation of mTORC1 is sufficient to accelerate aging in skeletal muscle. We aimed to address this question using an inducible model of muscle‐specific knockout of *Depdc5*, which encodes the protein DEP domain containing 5 (DEPDC5), a negative regulator of mTORC1. In 27‐month‐old mice, *Depdc5* knockout induced a robust upregulation of inflammatory and immune‐related genes, accompanied by alterations in metabolic and angiogenic pathways. Additionally, *Depdc5* knockout mice exhibited an increase in TNF‐α protein expression. However, these transcriptional and protein changes were not associated with an altered macrophage phenotype or with changes in muscle capillary density. Dietary rapamycin partially reversed mTORC1 signaling and some of the transcriptional changes, including those related to angiogenesis, but did not suppress inflammation‐related transcripts or protein expression. These findings demonstrate that constitutive activation of mTORC1 is sufficient to drive pro‐inflammatory changes in aged skeletal muscle, adding to the inflammation that already occurs with aging.

## Graphical abstract

mTORC1 activity increases with aging and may contribute to skeletal muscle decline. In aged mice, muscle‐specific *Depdc5* knockout activated mTORC1, increased inflammatory and immune‐related mRNA expression and TNF‐α protein without altering macrophage phenotype or capillary density. Rapamycin partially reversed mTORC1 signaling but not inflammation.

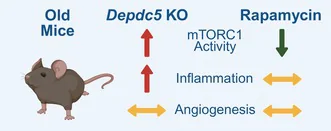

## 1 Introduction

The mechanistic target of rapamycin complex 1 (mTORC1) is a central regulator of growth, metabolism, and protein turnover (Sabatini et al. 1994). mTORC1 activity in skeletal muscle increases in the fed state and is reduced during fasting, leading to oscillating activity that is central to cellular homeostasis (Goul et al. 2023). However, during aging, basal mTORC1 activity is increased and is thought to play a critical role in age‐related declines in muscle health (Markofski et al. 2015). While it is known that pharmacological inhibition of mTORC1 improves muscle health, along with overall healthspan and lifespan (Harrison et al. 2009; Ham et al. 2022), it is less clear whether increased mTORC1 activity during aging is sufficient to accelerate aging phenotypes in skeletal muscle. We previously reported that muscle‐specific deletion of *Depdc5*, which encodes the protein DEP domain containing 5 (DEPDC5), increases mTORC1 signaling, associated with mild hypertrophy but no improvements in muscle strength or exercise capacity (Graber et al. 2019). These findings raise the question of whether chronic mTORC1 activation (like that observed in aging) might induce maladaptive changes in muscle that counteract any of the potential benefits of sustained anabolism.

Given the well‐established link between aging and low‐grade chronic inflammation, often referred to as “inflammaging” (Franceschi et al. 2000), we hypothesized that skeletal muscle from *Depdc5* knockout (KO) mice would exhibit an inflammatory transcriptional profile resembling aged muscle. Previously published work in 9‐month‐old mice showed that *Depdc5* knockout increased gene expression related to inflammation and the immune response, increased protein expression of the proinflammatory cytokines IL‐6 and TNF‐α, and shifted macrophages toward an M2‐like phenotype (Marchant et al. 2026). In the present study, we sought to extend these findings to 27‐month‐old mice, combining inducible *Depdc5* knockout with dietary rapamycin treatment to determine whether rapamycin could reverse inflammatory gene expression signatures driven by constitutively active mTORC1 in aged skeletal muscle. To further assess the relevance of these findings to physiological aging, we also examined inflammatory transcriptional signatures in skeletal muscle from young and aged common marmosets, including an aged cohort treated chronically with rapamycin.

Together, our results demonstrate that constitutively active mTORC1 is sufficient to drive pro‐inflammatory gene expression and increase protein expression of TNF‐α in aged skeletal muscle. However, this inflammatory signature was not reversed by rapamycin in *Depdc5* knockout mice and was not accompanied by changes in macrophage infiltration or capillary density. These findings provide new insight into the role of mTORC1 in skeletal muscle aging and inflammation.

## 2 Results

We recently published work showing that 9‐month‐old *Depdc5* knockout mice have elevated gene expression related to various biological processes, including immune function, cell division, and metabolic stress. We also observed a reduction in various metabolism‐related pathways and in gene expression related to angiogenesis. Furthermore, these mice exhibited an increase in protein expression of IL‐6 and TNF‐α, along with a shift in macrophages toward an M2‐like phenotype (Marchant et al. 2026).

Due to these previously reported results in adult mice, we hypothesized that *Depdc5* KO would exacerbate inflammation in aged mice. We also hypothesized that this inflammation could be reversed by pharmacological inhibition of mTORC1 with rapamycin. To test these hypotheses, we knocked out *Depdc5* in 22‐month‐old female and male mice. We chose this timepoint because C57BL/6j mice appear to be particularly susceptible around this age to declines in overall health and to death (Zhang et al. 2014). As such, we hypothesized that this age would increase their susceptibility to mTORC1‐driven changes in inflammation. Following tamoxifen‐induced knockout of *Depdc5* at 22 months of age, we aged the mice for 5 more months and collected tissues at 27‐months‐old. During the 5‐month span after tamoxifen administration, KO mice were either fed a control chow diet or a diet with rapamycin incorporated into the chow at a concentration of 14 ppm (Figure 1A). At the protein level, we observed clear increases in the phosphorylation of downstream targets of mTORC1 with *Depdc5* knockout, which was partially reversed by rapamycin (Figure 1B–I). Additionally, we observed altered expression of some autophagy proteins with KO, which were partially reversed by rapamycin treatment (Supplemental Figure 1).

**FIGURE 1:**
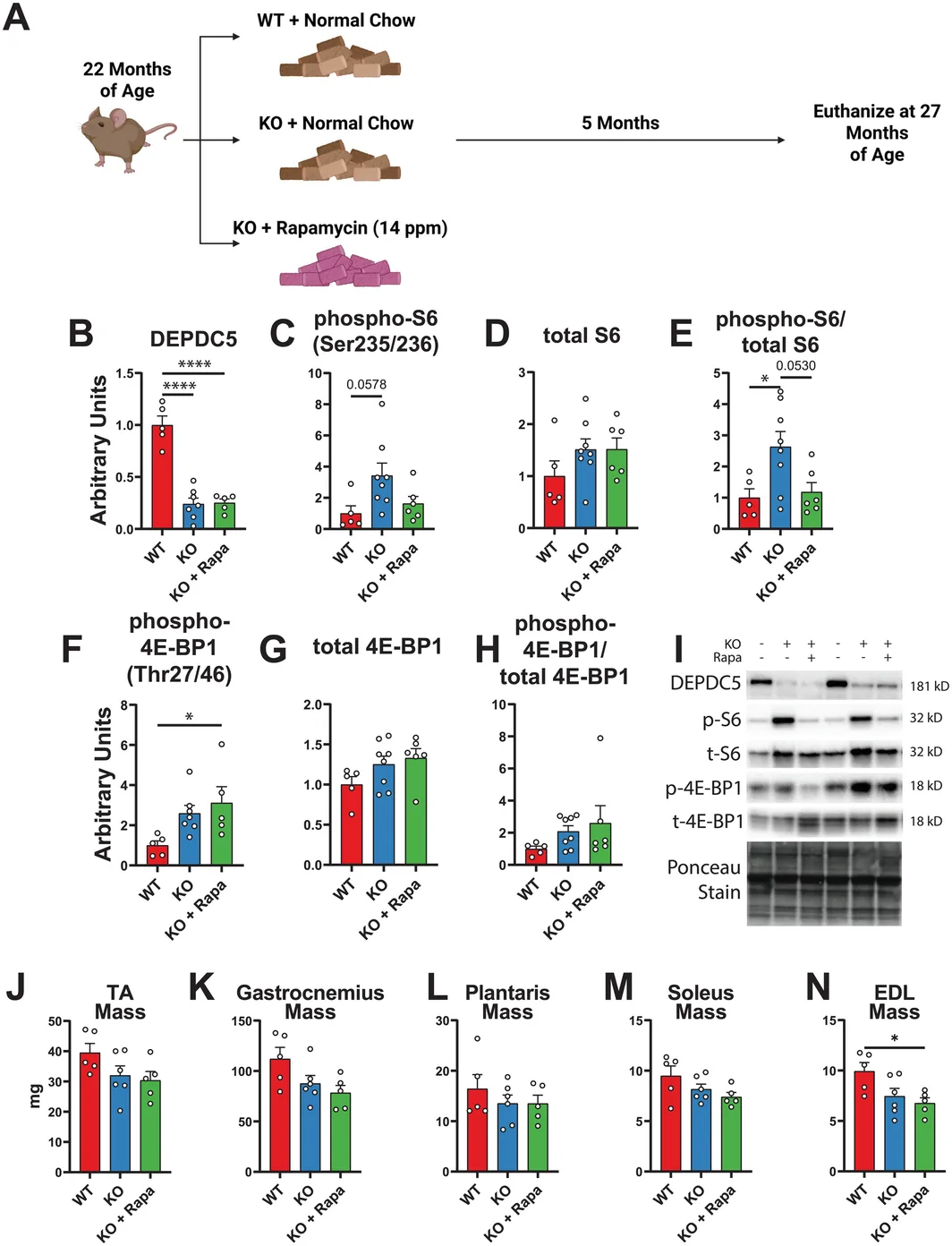
*Depdc5* KO increases phosphorylation of downstream targets of mTORC1 in aged mice, which is partially restored by rapamycin. (A) Study Schematic. (B–H) Relative quantification of western blots (*n* = 5–7). All westerns are normalized to total protein (Ponceau S), with WT set arbitrarily to a value of one. (I) Representative western blots and Ponceau S stain. (J–N) Skeletal muscle weights (*n* = 5–6). * indicates *p* < 0.05, **** indicates *p* < 0.0001. All data are represented as means ± SEM, along with individual data points.

To determine the effects of *Depdc5* KO on muscle size, we collected and weighed various skeletal muscles from the hindlimb during tissue collection. We observed a statistical difference between groups only in the extensor digitorum longus (EDL) muscle, determined by a one‐way ANOVA, while the remaining muscles (soleus, plantaris, gastrocnemius, tibialis anterior) did not reach significance (Figure 1J–N). However, we also performed a two‐way ANOVA, considering each muscle to be a repeated measure. In this case, the effect of treatment approached significance (*p* = 0.067). Post hoc analysis in the EDL revealed a significant difference between the WT and KO + Rapa groups (*p* < 0.05). Overall, these findings suggest that changes in muscle size following *Depdc5* KO and rapamycin were minimal. However, it is possible that with more animals, we might observe statistical differences due to *Depdc5* KO.

We then performed mRNA sequencing on gastrocnemius muscles and observed large changes in gene expression due to *Depdc5* KO, as well as large changes in KO animals when treated with rapamycin (Figure 2A–C). Of the 238 genes that were differentially regulated in KO mice compared to WT, 89 of those genes were reversed by rapamycin administration (Figure 2D,E, Supplemental Tables 1 and 2). GSEA analysis indicated that KO mice had increased expression related to inflammation and the immune response, similar to what we previously reported in 9‐month‐old mice (Marchant et al. 2026). Interestingly, the inflammatory profile was not strongly reversed by rapamycin, even though rapamycin is known to be an immunosuppressant (Dumont et al. 1990). We also observed a reduction in gene expression related to angiogenesis following *Depdc5* knockout, which was reversed by rapamycin treatment (Figure 2F–I, Supplemental Tables 3 and 4).

**FIGURE 2:**
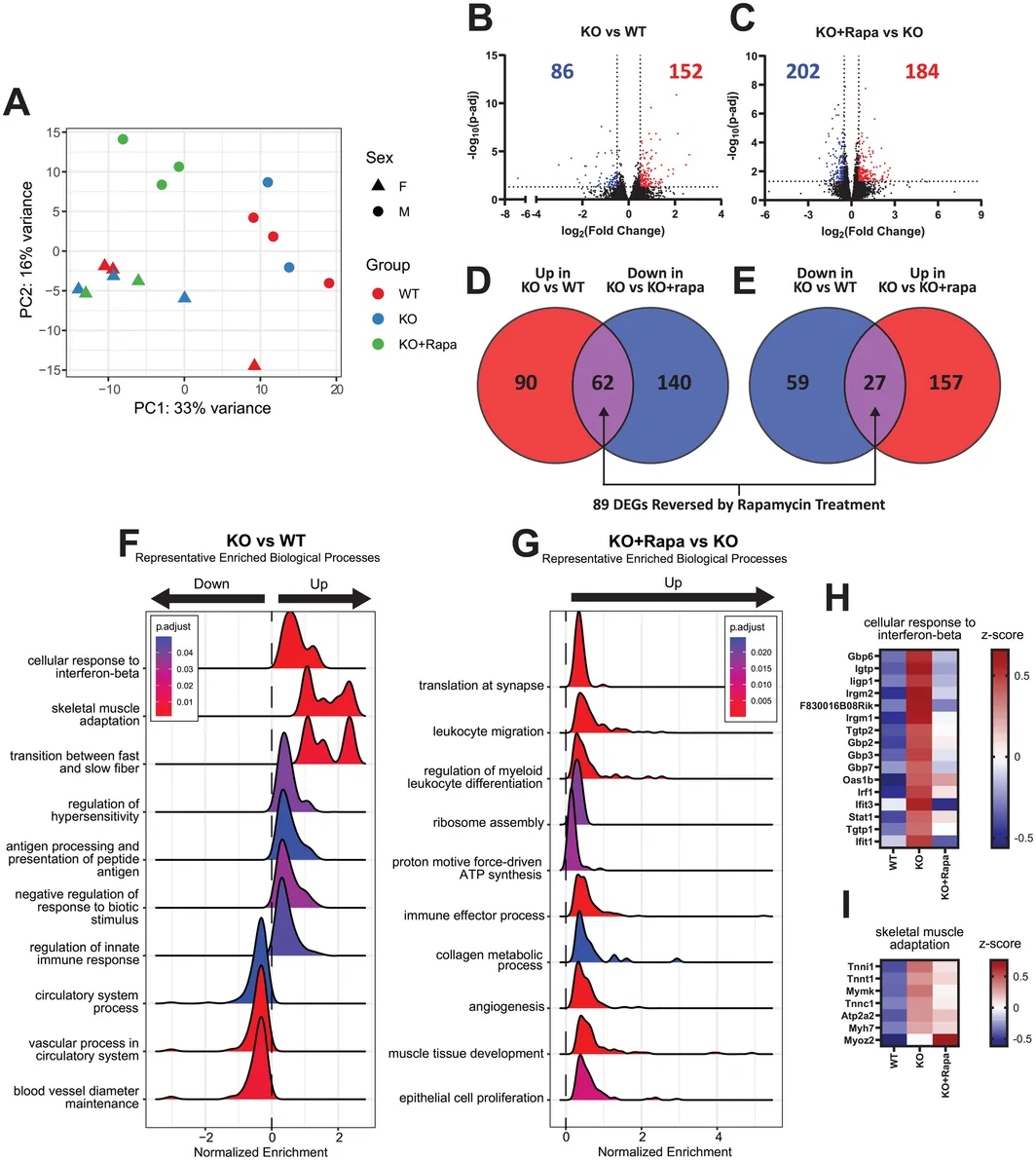
*Depdc5* KO increases gene expression related to inflammation, the immune response, and muscle adaptation, and decreases expression related to angiogenesis, which is partially reversed by rapamycin. (A) PCA plot of RNAseq data (*n* = 5–6). (B, C) Volcano plots depicting differentially expressed genes in KO vs. WT and KO + Rapa vs. KO mice. (D, E) Venn diagrams of differentially expressed genes with KO or rapamycin. (F, G) Ridge plots of representative biological processes differentially regulated as shown by Gene Set Enrichment Analysis. (H, I) Heatmaps of 2 representative pathways altered by KO, with partial reversal by rapamycin.

Given the effect of mTORC1‐driven increases in inflammation, we investigated whether *Depdc5* knockout altered inflammation markers at the protein level. We did not observe a significant change in IL‐6 protein expression (Figure 3A). However, there was a significant increase in total TNF‐α in KO mice, which was not reversed by rapamycin (Figure 3B). There was also an apparent increase in the 17 kD TNF‐α band, which is the cleavage product and actively secreted cytokine (Figure 3C). Interestingly, we observed a high level of variability in IL‐6 and TNF‐α protein. As a result, we sought to determine if the variability and trend were consistent with mRNA expression of the corresponding genes. We saw no differences between groups with respect to mRNA levels of *Il6* and *Tnf*, suggesting that the protein expression differences are regulated in a post‐transcriptional manner (Supplemental Figure 2). We next wanted to determine if the increase in TNF‐α and inflammation‐related genes with KO was associated with increased infiltration of macrophages in skeletal muscle. We measured total macrophage content and M2‐like macrophage content via immunohistochemistry, using F4/80 as a marker of all macrophages, and CD206 as a marker of M2‐like macrophages. Despite the observed changes in inflammation markers at the mRNA and protein levels, we saw no changes in macrophage number or phenotype in our immunohistochemical measures (Figure 3E–H). These findings are contrary to our previous report in 9‐month‐old mice, which exhibited a shift toward an M2‐like phenotype with *Depdc5* knockout (Marchant et al. 2026). Next, we sought to determine if the observed changes in mRNA expression related to angiogenesis were associated with alterations in capillary density in skeletal muscle. Despite clear examples of reduced endothelial cell‐related mRNA expression with KO (i.e., *Pecam1*, which encodes the endothelial cell marker CD31), and reversal with rapamycin administration (Figures 2F,G and 4A), we saw no changes in capillary density with our treatments (Figure 4B–D). This finding indicates that in aged mice, changes in mTORC1 activity are not sufficient to drive changes in capillarization, despite altered gene expression.

**FIGURE 3:**
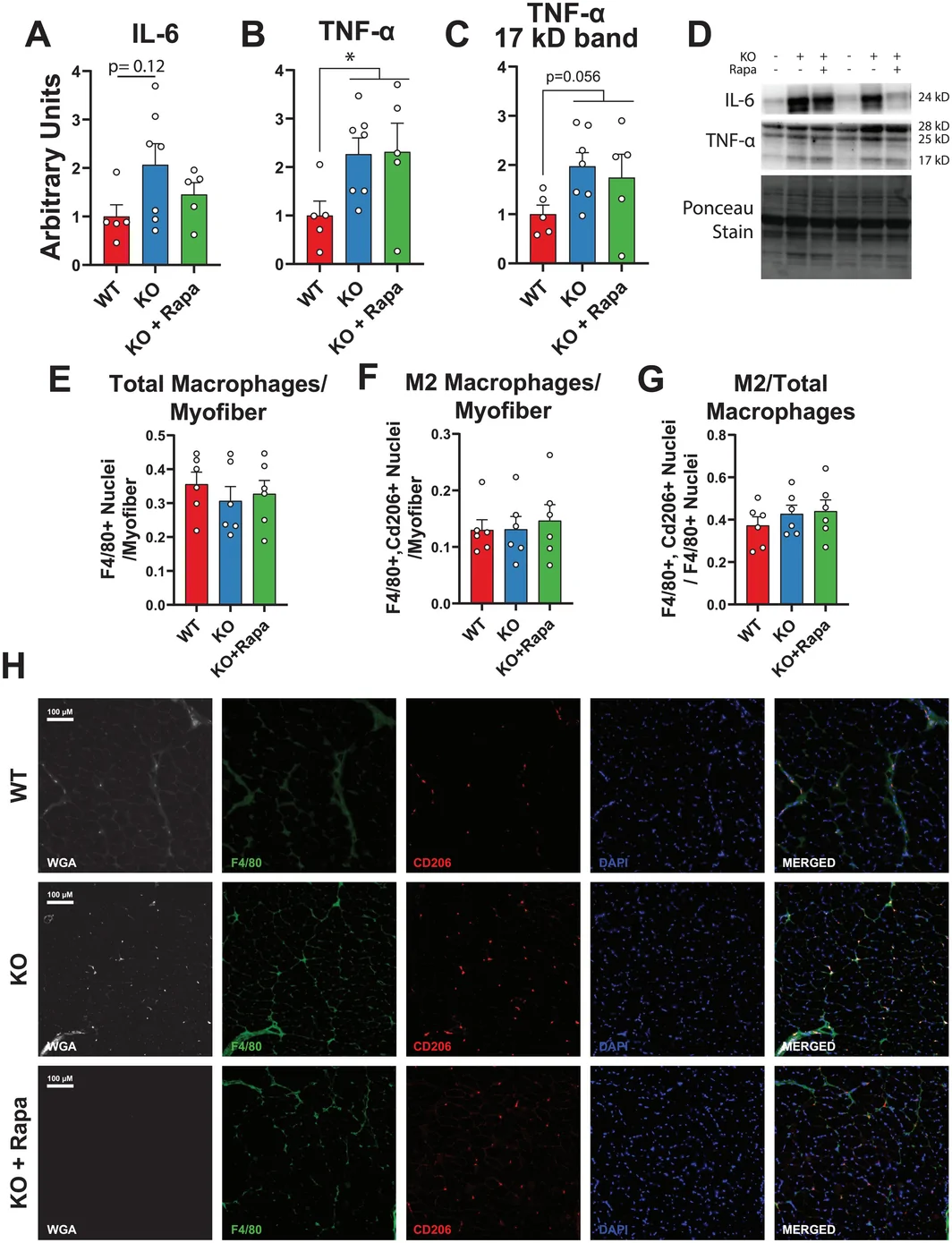
*Depdc5* KO increases TNF‐α protein expression but does not alter macrophage infiltration or phenotype in muscle sections. (A–C) Relative quantification of western blots (*n* = 5–7). All westerns are normalized to total protein (Ponceau S), with WT set arbitrarily to a value of one. (D) Representative western blots and Ponceau S stain. (E, F) Quantification of total and M2 macrophages relative to myofiber number (*n* = 6). (G) Ratio of M2/total macrophages. (H) Representative images of IHC in muscle sections. * indicates *p* < 0.05. All data are represented as means ± SEM, along with individual data points.

**FIGURE 4:**
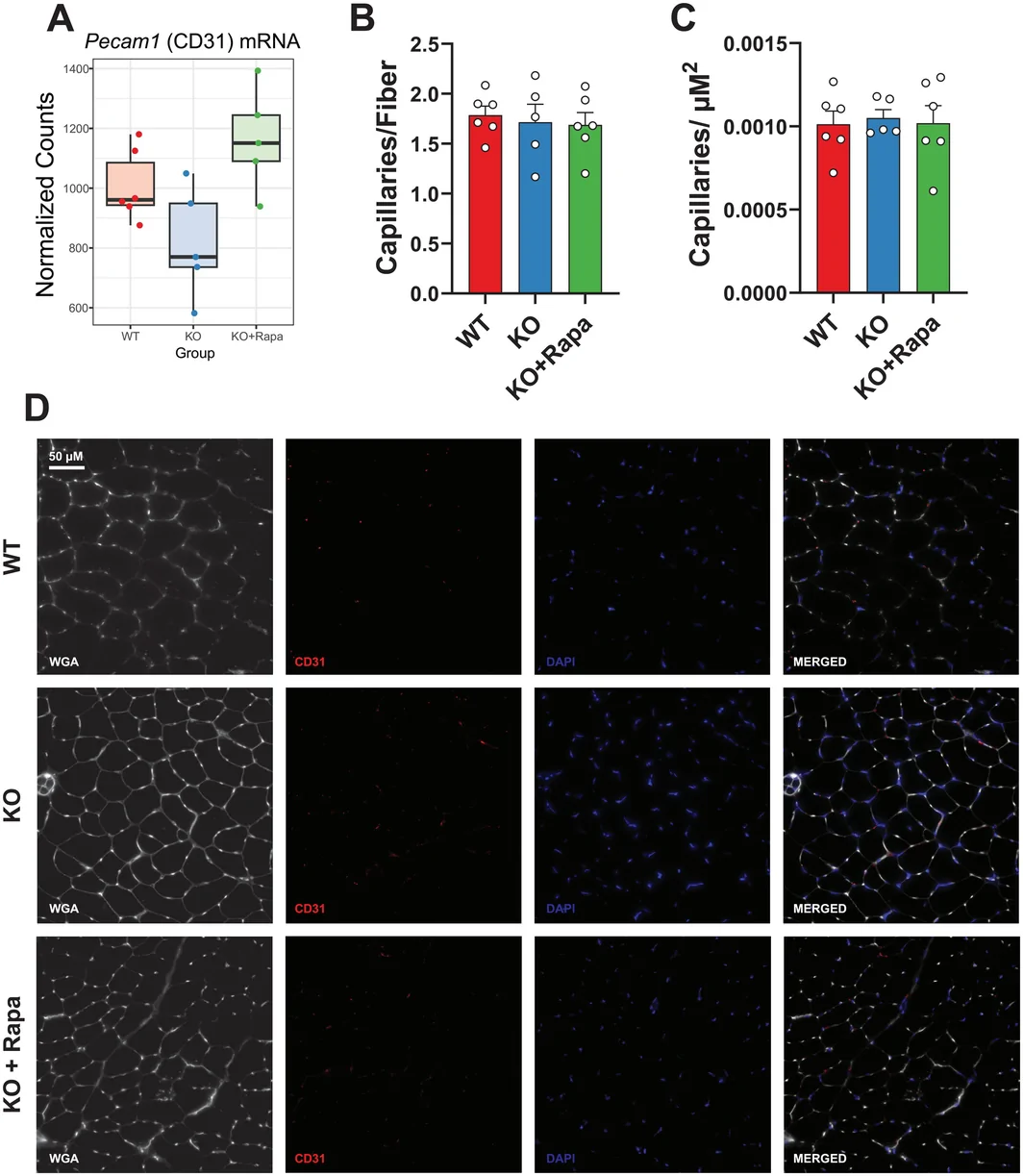
*Depdc5* KO does not alter capillary density in skeletal muscle sections. (A) *Pecam1* (CD31) mRNA expression from RNAseq data. (B, C) Quantification of capillary density per muscle fiber and per μM^2^ (*n* = 6). (D) Representative images of IHC in muscle sections. All data are represented as means ± SEM, along with individual data points.

To better understand the effect of physiological aging and mTORC1 inhibition on skeletal muscle inflammation, we performed transcriptomic analyzes of skeletal muscle from young and aged common marmosets, including an aged cohort treated chronically with rapamycin. Consistent with our finding that mTORC1 activation exacerbates inflammation, aging was associated with increased expression of genes related to inflammation and the immune response, and rapamycin treatment reduced inflammatory pathway expression (Supplemental Figure 3, Supplemental Tables 5–8). Together, these findings provide additional evidence that inflammatory transcriptional changes accompany physiological aging, and that mTORC1 inhibition can ameliorate inflammation in the context of aging.

## 3 Discussion

It is well‐known that reducing mTORC1 activity is a potential target for improving lifespan and healthspan in mice (Harrison et al. 2009; Miller et al. 2011; Zhang et al. 2017, 2024). Additionally, reducing mTORC1 activity is often associated with reduced inflammation (Ham et al. 2022; Zhang et al. 2024). However, reduced mTORC1 activity is not always associated with reduced inflammation, indicating that the role of mTORC1 is nuanced (Correia‐Melo et al. 2019). In skeletal muscle, it is also well‐known that mTORC1 activity increases with age, suggesting it may be a potential driver of age‐related changes in muscle health (Markofski et al. 2015). Treatment with an mTORC1 inhibitor has been shown to reduce mTORC1 signaling and to counteract age‐related declines in muscle mass in rats (Joseph et al. 2019). Additionally, exercise training in mice has been shown to inhibit mTORC1 activity and thus improve skeletal muscle health in aging (Choy et al. 2025). Most prior studies have focused solely on mTORC1 inhibition as a potential target for improved health and longevity. Few studies have tested whether increasing mTORC1 activity is sufficient to drive or exacerbate aging phenotypes.

This work provides new insight into the role of mTORC1 in inflammation and aging skeletal muscle. The hypothesis that *Depdc5* KO would drive inflammation is plausible, as previous work has shown that simultaneous germline knockout of *Tsc1* and *Depdc5* (both negative regulators of mTORC1 activity) is sufficient to drive early skeletal muscle myopathy, cardiac and diaphragm dysfunction, and inhibition of autophagy, accompanied by large increases in muscle inflammation (Cho et al. 2022). Additionally, *Tsc1* knockout alone is sufficient to drive skeletal muscle atrophy in 12‐month‐old mice, associated with impaired autophagy, and is partially reversible with rapamycin treatment (Castets et al. 2013). We have also recently reported that *Depdc5* KO in adult mice can drive inflammation (Marchant et al. 2026). However, this work provides additional evidence that mTORC1 activation can drive inflammation even in aged skeletal muscle.

### 3.1 Depdc5 Knockout as a Method for Activating mTORC1

In this work, we decided to test the effects of mTORC1 hyperactivation in skeletal muscle by knocking out the *Depdc5* gene in skeletal muscle. *Depdc5*, which encodes the protein DEPDC5, is one of the components of the GATOR1 protein complex, which negatively regulates mTORC1 activity via its interaction with the Rag GTPases (Bar‐Peled et al. 2013). GATOR1 becomes active during periods of amino acid insufficiency, which leads to inhibition of mTORC1 activity. Therefore, *Depdc5* knockout activates mTORC1 via a nutrient sensing pathway. There is no single defined mechanism for why mTORC1 is activated in aging skeletal muscle. Thus, it is important to investigate how different models of mTORC1 activation affect skeletal muscle health in the context of aging and metabolic disease. We do not suggest that *Depdc5* KO is the only or the best model for activating mTORC1, as there are many ways to accomplish this, including established models such as Tsc1 KO, which drives a pronounced aging and metabolic phenotype (Castets et al. 2013; Guridi et al. 2016). However, given that *Depdc5* knockout appears to have a distinct phenotype from *Tsc1* knockout, this model provides important information that complements what has been gained from other existing models. Furthermore, the amino acid‐sensing pathway, of which DEPDC5 is a part, is relevant to aging, given that amino acid restriction has been shown to improve healthspan and lifespan in rodents and other model organisms (Richardson et al. 2021; Zhang et al. 2023).

### 3.2 Inflammation Induced by mTORC1 Activation in the Context of Aging

Aging itself is associated with increased inflammation in skeletal muscle, and this age‐associated inflammatory state is an important component of skeletal muscle aging and functional decline (Kedlian et al. 2024; Merritt et al. 2013; Caldow et al. 2013). Thus, our finding that *Depdc5* KO increased inflammatory gene and protein expression in 27‐month‐old mice could be interpreted as an additional inflammatory stimulus superimposed on an already aged muscle environment. Indeed, studies in aged mice have demonstrated increased inflammatory signaling in skeletal muscle, while recent work has further linked age‐associated inflammation to increased mTORC1 activity (Zhang et al. 2022; Cao et al. 2025). Our findings extend these observations by demonstrating that experimentally increasing mTORC1 activity above the level present in age‐matched controls is sufficient to further increase inflammatory signaling in aged skeletal muscle. This distinction is important because it suggests that elevated mTORC1 activity may contribute to the amplification of age‐associated inflammation rather than being the sole cause of the inflammatory state that develops during physiological aging. At the same time, the absence of substantial changes in muscle size, capillary density, or macrophage abundance with *Depdc5* KO indicates that this additional inflammatory signaling may be insufficient alone to exacerbate the broader structural consequences of physiological aging.

Given the immunomodulatory actions of rapamycin (Abraham and Wiederrecht 1996; Eng et al. 1991; Kahan et al. 1991), we expected to also observe a full or partial reversal of inflammatory genes with rapamycin treatment. However, we saw no clear reduction in inflammatory genes or proteins with rapamycin treatment in KO mice. This is interesting, since we observed clear reductions in inflammation in marmosets treated with rapamycin, which further suggests that mTORC1 plays a role in age‐related muscle inflammation. Part of the reason why rapamycin may not have reversed inflammation in KO mice is that rapamycin only partially inhibited mTORC1, in that 4E‐BP1 phosphorylation was not reduced by rapamycin. This finding is largely consistent with other data suggesting that rapamycin may not equally prevent all mTORC1 targets from being phosphorylated (Choo et al. 2008). Additionally, we expected that part of the reason for increased inflammation with *Depdc5* knockout would be a change in macrophage infiltration in skeletal muscle, or a shift in the macrophage phenotype to be more pro‐inflammatory. Contrary to our hypothesis, we did not see an effect of *Depdc5* KO or of rapamycin on macrophage number or phenotype in aged mice. This is different than our previous finding that there was a significant shift toward M2‐like macrophage polarization in 9‐month‐old KO mice compared to controls (Marchant et al. 2026). Additionally, mTORC1 inhibition by rapamycin has been shown to cause a shift toward a more M1‐like macrophage phenotype (Byles et al. 2013; Mercalli et al. 2013). Interestingly, it has been reported that aging decreases the M1:M2 ratio in mice (Liu et al. 2023), but this is largely the opposite of what typically occurs in humans (Cui et al. 2019; Ahmadi et al. 2022). Part of this discrepancy may be because the classification of macrophages into simple M1 or M2 categories is an oversimplification of the various roles and niches these cells occupy in physiology (Krasniewski et al. 2022). Regardless, in aged mice, we did not observe an effect of *Depdc5* KO or rapamycin on macrophage number or phenotype.

### 3.3 Angiogenesis and Capillary Density

We observed that angiogenic and endothelial cell‐related gene expression was reduced in *Depdc5* KO mice compared to WT, which was consistent with our previous observations in 9‐month‐old mice (Marchant et al. 2026). Additionally, rapamycin increased angiogenic gene expression, suggesting that mTORC1 may be a regulator of changes in angiogenesis. Despite these clear changes in angiogenic markers, we did not observe a change in capillary density between KO and WT animals, nor did we observe an effect of rapamycin treatment on capillary density. It is possible that the duration of the intervention was insufficient to drive changes in capillary density, or that the gene expression was more related to endothelial function, and not necessarily to capillary content. In human subjects, it has been frequently reported that capillary density declines with age, and that these declines are strongly associated with decrements in muscle function and responsiveness to exercise training (Groen et al. 2014; Moro et al. 2019). However, it should be noted that this finding is not always consistent and may be partially the result of limitations in analytical techniques (Frydenholm et al. 2026). Regardless, our findings here suggest that constitutive activation of mTORC1 may not be sufficient alone to reduce capillary density.

### 3.4 Conclusion and Limitations

Our findings demonstrate that constitutive activation of mTORC1 in skeletal muscle is sufficient to induce a robust pro‐inflammatory transcriptional program in aged skeletal muscle. Several limitations of this work should be considered. First, our assessment of immune cell dynamics relied primarily on immunohistochemical markers of macrophage abundance and polarization, which may not fully capture functional heterogeneity or subtle shifts in immune cell subsets. Next, although rapamycin partially suppressed mTORC1 signaling, incomplete inhibition of downstream targets (e.g., 4E‐BP1 phosphorylation) limits our ability to determine whether more complete mTORC1 suppression would have yielded different inflammatory outcomes. Additionally, this study was not powered to detect sex differences. However, it is possible that sexual dimorphism in the response to *Depdc5* KO exists. It is also possible that there are mTORC1‐independent effects related to *Depdc5* knockout, as DEPDC5 has been reported to have an mTORC1‐independent role in the brain (Swaminathan et al. 2018). Another important consideration is whether the additional inflammatory signaling induced by *Depdc5* KO in aged muscle has functional consequences. Although we observed minimal effects of *Depdc5* KO on skeletal muscle mass, muscle mass alone does not necessarily reflect muscle quality or function. Indeed, our previous work using the same *Depdc5* KO model demonstrated that increased mTORC1 activity and muscle hypertrophy were not accompanied by improvements in muscle contractile or physical function (Graber et al. 2019; Marchant et al. 2026). However, the present study does not include measures of muscle function, and therefore, cannot determine whether the additional inflammatory phenotype observed in 27‐month‐old KO mice is associated with impaired muscle strength, contractile function, or physical performance. This is an important limitation when interpreting the absence of a clear muscle atrophy phenotype in the KO mice. Future studies incorporating measures of muscle strength, contractile function, and physical performance will be necessary to determine whether the inflammatory phenotype induced by *Depdc5* KO contributes to functional decline in aged skeletal muscle. We also did not perform direct comparisons between different skeletal muscles, which limits our ability to determine if *Depdc5* KO alters inflammation in a fiber type‐specific manner. Finally, the independent effects of age and genotype cannot be fully disentangled in the current experimental design, and future studies directly comparing young and aged knockout mice under identical conditions will be necessary to address this question. Despite these limitations, this work provides important evidence that elevated mTORC1 drives inflammatory gene expression in skeletal muscle in the context of aging.

## 4 Methods

### 4.1 Mouse Experiments

*Depdc5* KO mice and floxed controls were generated as previously described (Graber et al. 2019), and genotypes were determined by qPCR. Mice were housed on a 12:12 h light: dark cycle. All procedures were approved either by the UT Medical Branch or the UT Health San Antonio Institutional Animal Care and Use Committees. Beginning at 22 months of age, all KO mice were administered tamoxifen every other day for 5 days (75 mg/kg, dissolved in sunflower oil, administered via intraperitoneal injection), followed by another injection 3 days later, and one every 28 days until the end of the study (*n* = 6–8 per group, with even numbers of males and females). Following administration of tamoxifen, mice were fed either a control diet (LabDiet #5001) or diet containing 14 ppm microencapsulated rapamycin, which was obtained from Emtora Biosciences (formerly Rapamycin Holdings). Mice continued these diets for 5 months, at which point mice were euthanized for tissue collection. At the time of euthanasia, skeletal muscles were flash frozen for RNA or protein extraction, or were embedded in optimal cutting medium (OCT; Fisher Scientific; 23‐730‐571) and frozen in isopentane cooled in liquid nitrogen for tissue sectioning and immunohistochemistry (IHC). See Figure 1A for a study schematic.

### 4.2 Marmoset Experiment

All marmosets used in this research were housed at the Barshop Institute for Longevity and Aging Studies at UT Health San Antonio, and this research was approved by the UT Health San Antonio Institutional Animal Care and Use Committee. Additionally, this experiment was conducted in compliance with the US Public Health Service's Policy on Humane Care and Use of Laboratory Animals and the Guide for the Care and Use of Laboratory Animals and adhered to the American Society of Primatologists (ASP) principles for the ethical treatment of non‐human primates. Animals used in this study were selected based on age (Table 1) as well as a record of relatively good health as assessed by veterinary examination. Rapamycin encapsulated in Eudragit (and Eudragit alone) was purchased from Rapamycin Holdings (now Emtora; San Antonio TX). Marmosets were trained to receive oral dosing of rapamycin (or eudragit alone) mixed into yogurt on a weekly basis. Marmosets were treated once daily with a dose of rapamycin roughly equivalent to 1 mg rapamycin/kg body weight. Animals were dosed Monday–Friday with dose received between 08:00 and 10:00 but not dosed on Saturday or Sunday. Marmosets were part of a long‐term study to assess the effect of rapamycin on marmoset longevity, and all muscle samples were collected at end‐of‐life necropsy.

**TABLE 1:** Marmoset ages (years).

Group	Females, *n* = 3**	Males, *n* = 3****	Both, *n* = 6****
Young	4.1 ± 0.4	4.2 ± 0.3	4.2 ± 0.3
Old	11.4 ± 0.4	12.7 ± 0.5	12.1 ± 0.4
Old + Rapa	11.7 ± 1.0	13.0 ± 0.5	12.4 ± 0.6

*Note:* ** indicates ANOVA with *p* < 0.01, **** indicates *p* < 0.0001.

### 4.3 Western Blotting

Quadriceps muscles were homogenized at a 5% concentration in ice‐cold lysis buffer (50 mM Tris HCl, 250 mM mannitol, 1 mM EDTA, 1 mM EGTA, 1% Triton‐X 100, 1 mM DTT) supplemented with protease and phosphatase inhibitors (ThermoFisher, 78,442), using a glass‐on‐glass homogenizer. Samples were centrifuged for 10 min at 16,000 rcf and 4°C. Total protein concentration was measured in supernatants using bicinchoninic acid methodology (ThermoFisher, 23,225) and diluted to a protein concentration of 2 mg/mL in Laemmli buffer. Proteins were separated via electrophoresis on 4%–15% polyacrylamide gels (BioRad, 5,671,084) in Tris/Glycine/SDS electrophoresis buffer (BioRad, 1610772EDU). Thirty micrograms of total protein were loaded in each well. Following electrophoresis, proteins were transferred to PVDF membranes at 100 V for 60 min in ice‐cold transfer buffer (BioRad, 1610771EDU). Following the protein transfer, the membranes were stained for total protein to ensure equal loading using Ponceau S BioReagent (ThermoFisher, A40000279). Each membrane was incubated overnight at 4°C in 5% BSA in tris‐buffered saline, 0.1% TWEEN 20 (TBST), followed by a 4‐h application of primary antibody at RT in 1% BSA in TBST. For a list of antibodies used, see Table 2. Membranes were washed in TBST and subsequently incubated for 60 min at RT in HRP‐linked secondary antibody (Table 2), diluted in 1% BSA in TBST. Membranes were again washed in TBST, incubated for 5 min at RT in SuperSignal West Femto Maximum Sensitivity Substrate (ThermoFisher, #34094), and imaged with various exposure durations (FluorChem M, Protein Simple). Optical density of each band was assessed using ImageJ, and all band values were normalized to total protein measured via Ponceau S staining.

**TABLE 2:** List of reagents.

Reagent	Concentration; application	Vendor; catalog number
Anti‐DEPDC5 antibody [EPR20497‐23]	1:500; Western	Abcam; ab213181
Phospho‐S6 Ribosomal Protein (Ser235/236) Antibody	1:1000; Western	Cell Signaling Technology; 2211
S6 Ribosomal Protein (5G10) Rabbit Monoclonal Antibody	1:1000; Western	Cell Signaling Technology; 2217
Phospho‐4E‐BP1 (Thr37/46) (236B4) Rabbit Monoclonal Antibody	1:1000; Western	Cell Signaling Technology; 2855
4E‐BP1 Antibody	1:1000; Western	Cell Signaling Technology; 9452
IL‐6 (D5W4V) Rabbit Monoclonal Antibody	1:500; Western	Cell Signaling Technology; 12,912
TNF‐alpha (D2D4) Rabbit Monoclonal Antibody	1:500; Western	Cell Signaling Technology; 11,948
SQSTM1/p62 Antibody	1:1000; Western	Cell Signaling Technology; 5114
Parkin Antibody	1:1000; Western	Cell Signaling Technology; 2132
Beclin‐1 Antibody	1:1000; Western	Cell Signaling Technology; 3738
Phospho‐ULK1 (Ser757) (D7O6U) Rabbit Monoclonal Antibody	1:1000; Western	Cell Signaling Technology; 14,202
p53 (1C12) Mouse Monoclonal Antibody	1:1000; Western	Cell Signaling Technology; 2524
Purified Mouse Anti‐p21 Antibody	1:500; Western	BD Biosciences; 556,430
anti‐rabbit IgG HRP‐linked secondary antibody	1:20000; Western	Cell Signaling Technology; 7074
anti‐mouse IgG HRP‐linked secondary antibody	1:20000; Western	Cell Signaling Technology; 7076
Rat anti‐mouse F4/80 antibody	1:200; IHC	AbD Serotec; MCA497GA
Goat anti‐CD206 antibody	1:200; IHC	R&D Systems; AF2534
biotinylated goat anti‐rat IgG (H + L) secondary	1:1000; IHC	Invitrogen; PI31830
rabbit anti‐goat IgG secondary antibody	1:500; IHC	Thermofisher, 31,402
wheat germ agglutinin AF647	1:50; IHC	Thermofisher; 32,466
AF488 Tyramide Superboost	1:10; IHC	Thermofisher; B40932
streptavidin‐Texas Red conjugate	1:200; IHC	Vector Laboratories; SA‐5006
DAPI, Dilactate	1:10000; IHC	Thermofisher; D3571

### 4.4 mRNA Sequencing

Total RNA was extracted from gastrocnemius muscle from mice or mixed hindlimb muscle from marmosets using TRIzol reagent (Thermo Fisher Scientific) and quantified using a NanoDrop spectrophotometer. RNA quality was assessed using an Agilent TapeStation (Agilent Technologies, Santa Clara, CA), and samples with an RNA quality number (RQN) > 7 were used for mRNA‐seq library preparation. Approximately 500 ng of total RNA was used for stranded mRNA‐seq library preparation according to the NEB Directional mRNA‐seq sample preparation protocol (New England Biolabs, Ipswich, MA). Polyadenylated RNA was isolated using poly‐T oligo‐attached magnetic beads, fragmented using divalent cations at elevated temperature, and reverse transcribed using random primers. cDNA fragments were subjected to end repair, dA‐tailing, adapter ligation, purification, and PCR enrichment to generate sequencing libraries. Libraries were quantified and sequenced using an Illumina NovaSeq 6000 platform with 100‐bp paired‐end reads. Sequencing data were demultiplexed using bcl2fastq2 to generate FASTQ files, with an average sequencing depth of approximately 30 million reads per sample.

Raw sequencing reads were processed using Trimmomatic (Galaxy version 0.39 + galaxy2) through the Galaxy web platform (usegalaxy.org). Paired‐end reads were processed as separate forward and reverse‐read files. Adapter sequences were removed using the Illumina TruSeq3 paired‐end adapter set with the following parameters: maximum mismatch count of 2, palindrome clip threshold of 30, simple clip threshold of 10, and minimum adapter length of 8 nucleotides. Both reads of each paired‐end fragment were retained following adapter trimming. Remaining low‐quality sequence was removed using a sliding‐window approach with a window size of 4 bases and a minimum average Phred quality score of 20.

For mouse samples, trimmed reads were aligned to the mouse reference genome GRCm38 (mm10) using HISAT2 (Galaxy version 2.2.2 + galaxy0). Marmoset reads were aligned to the marmoset reference genome assembly GCF_011100555.1 using the same HISAT2 version. Alignment was performed using paired‐end library settings. Gene‐level read counts were subsequently generated from the aligned reads using featureCounts (Galaxy version 2.1.1 + galaxy1) counting paired‐end reads as a single fragment, excluding chimeric fragments. Prior to downstream differential expression and pathway analyzes, marmoset gene identifiers were converted to their corresponding human orthologs using the GRCh38 (hg38) human genome annotation.

For differential gene expression analysis, raw gene‐level count matrices were analyzed using DESeq2 version 1.44.0. Genes with a total count < 10 across all samples were excluded from downstream analysis. DESeq2 size‐factor normalization was then applied to account for differences in sequencing depth between samples. Differential expression was modeled using a design formula of ~ Sex + Group, with sex included as a covariate and Group representing the experimental condition. Pairwise differential expression was assessed for KO versus WT and rapamycin‐treated KO versus untreated KO comparisons in the mouse datasets, and for Old versus Young and rapamycin‐treated Old versus untreated Old comparisons in the marmoset dataset. Statistical significance was assessed using the Benjamini‐Hochberg procedure to control the false discovery rate. Normalized expression values were generated using the DESeq2 size‐factor normalization procedure.

Principal component analysis (PCA) was performed using the variance‐stabilizing transformation implemented in DESeq2 (vst; blind = TRUE), with the 500 genes exhibiting the greatest variance used for PCA visualization. PCA plots were generated using the Group and Sex variables to assess overall transcriptional relationships among samples.

Gene set enrichment analysis (GSEA) was performed using the clusterProfiler R package version 4.12.6 with the fgsea algorithm. For each differential expression comparison, genes with available Ensembl gene identifiers and non‐missing log2 fold‐change and adjusted *p* values were retained, and Ensembl identifiers were matched to the *Mus musculus* annotation database org.Mm.eg.db for mice, or to for marmosets. Genes were ranked according to their DESeq2 log2 fold‐change values and analyzed using the gseGO function for Gene Ontology Biological Process (GO:BP) enrichment. Gene sets containing 10–500 genes were included in the analysis. Statistical significance was assessed using a nominal *p*‐value cutoff of 0.05 with Benjamini‐Hochberg adjustment for multiple comparisons. To reduce redundancy among highly overlapping GO:BP terms, enriched gene sets were additionally processed using the simplify function in clusterProfiler with a semantic similarity cutoff of 0.5, retaining the term with the lowest adjusted *p* value among redundant terms.

Gene expression matrices and differential expression results for the mouse cohort (KO versus WT and rapamycin‐treated KO versus untreated KO), and marmoset cohort (Old versus Young and rapamycin‐treated Old versus untreated Old) are provided in Supplemental Tables 1–4.

### 4.5 Immunohistochemistry

Tibialis anterior muscles embedded in OCT were sectioned (7 μM‐thick) on a cryostat and air‐dried for 60 min. Sections were fixed for 8 min in acetone at −20°C. Methods for macrophage IHC have been described previously (Marchant et al. 2025). For capillary imaging and quantification, slides were first blocked using a streptavidin/biotin blocking kit (Vector Laboratories; SP‐2002), followed by 1 h of blocking in 2.5% normal horse serum at RT. Anti‐CD31 primary antibody and wheat germ agglutinin AF647 were applied overnight at 4°C in 2.5% normal horse serum. Slides were washed 3 times in PBS, then incubated in biotinylated secondary antibody for 1 h at RT in 2.5% normal horse serum. Slides were again washed 3 times in PBS, then incubated for 1 h at RT in streptavidin‐Texas Red conjugate in PBS. Slides were washed 3 times in PBS, and incubated for 10 min in DAPI, washed again in PBS. Coverslips were mounted using Vectashield mounting medium (Vector Laboratories; H‐1900). Slides were imaged at 40× using a Nikon Eclipse Ti inverted microscope. For macrophage images, 2 separate images were obtained for each sample and averaged. For capillary images, 3 separate images were obtained for each sample and averaged. For more detailed antibody and reagent information, see Table 2.

### 4.6 Statistics

For western blotting and immunohistochemistry, statistics were performed using GraphPad Prism. For 9‐month‐old mice, a Student's *T*‐Test was used to determine differences between WT and KO mice. For measures made in 27‐month‐old mice, a one‐way ANOVA was used. If *p*‐values from ANOVA analyzes were < 0.05, a Tukey HSD post hoc analysis was performed to determine pairwise differences. Unless otherwise specified, each measure is represented as means ± SEM.

## Author Contributions

E.D.M. conceptualized the study design and wrote the manuscript. B.B.R. supervised data collection and analysis. E.D.M., H.K., J.M., and Z.L. collected and analyzed the data. A.B.S. and B.B.R. provided financial support. B.B.R., A.B.S., and H.K. revised the manuscript.

## Funding

This work was supported by the National Institute on Aging, R56AG051267, P30AG013319, P30AG044271, T32AG021890, R01AG050797, U34AG068482. National Cancer Institute, P30CA054174. National Institutes of Health, S10OD030311. Cancer Prevention and Research Institute of Texas, RP220662.

## Conflicts of Interest

The authors declare no conflicts of interest.

## Supporting information


**Supplemental Figure 1:** *Depdc5* KO alters protein expression of autophagy markers, some of which are reversed by rapamycin treatment. (A–F) Relative quantification of western blots (*n* = 5–7). All westerns are normalized to total protein (Ponceau S), with WT set arbitrarily to a value of one. (G) Representative western blots and Ponceau S Stain. * indicates *p* < 0.05. ** indicates *p* < 0.01. All data are represented as means ± SEM, along with individual data points.
**Supplemental Figure 2:** Neither *Depdc5* KO nor rapamycin alter mRNA expression of *IL6* and *Tnf*. (A) *IL6* mRNA counts from RNAseq data. (B) *Tnf* mRNA counts from RNAseq data. All data are represented as means ± SEM, along with individual data points.
**Supplemental Figure 3:** Aged marmoset skeletal muscle exhibits increased inflammation markers and reduced protein translation relative to young marmosets, which is reversed by rapamycin treatment. (A) Ridge plots of representative biological processes differentially regulated in Old vs. Young or in Old + Rapa vs. Old marmosets as shown by Gene Set Enrichment Analysis. (B, C) Volcano plots depicting differentially expressed genes in Old vs. Young or in Old + Rapa vs. Old marmosets. (D) Heat maps of biological processes that are reversed with rapamycin treatment. Each group consists of 3 male and 3 female marmosets, and scaling represents normalized *z*‐scores.

**Data S1:** Supplementary figures 1–3, supplementary tables 1–8 and uncropped western blot images.

## Data Availability

The data that supports the findings of this study are available in the Supporting Information of this article.
