# Supplementary material for: Constitutive Activation of Skeletal Muscle mTORC1 Drives Pro‐Inflammatory Gene Expression in Skeletal Muscle of Aged Mice
Source: Aging Cell. 2026 Nov 6;25(11):e70805. doi: 10.1111/acel.70805 (PMC13741394; doi:10.1111/acel.70805)
Supplement: Supplementary file 1 [file ACEL-25-e70805-s002.pdf]

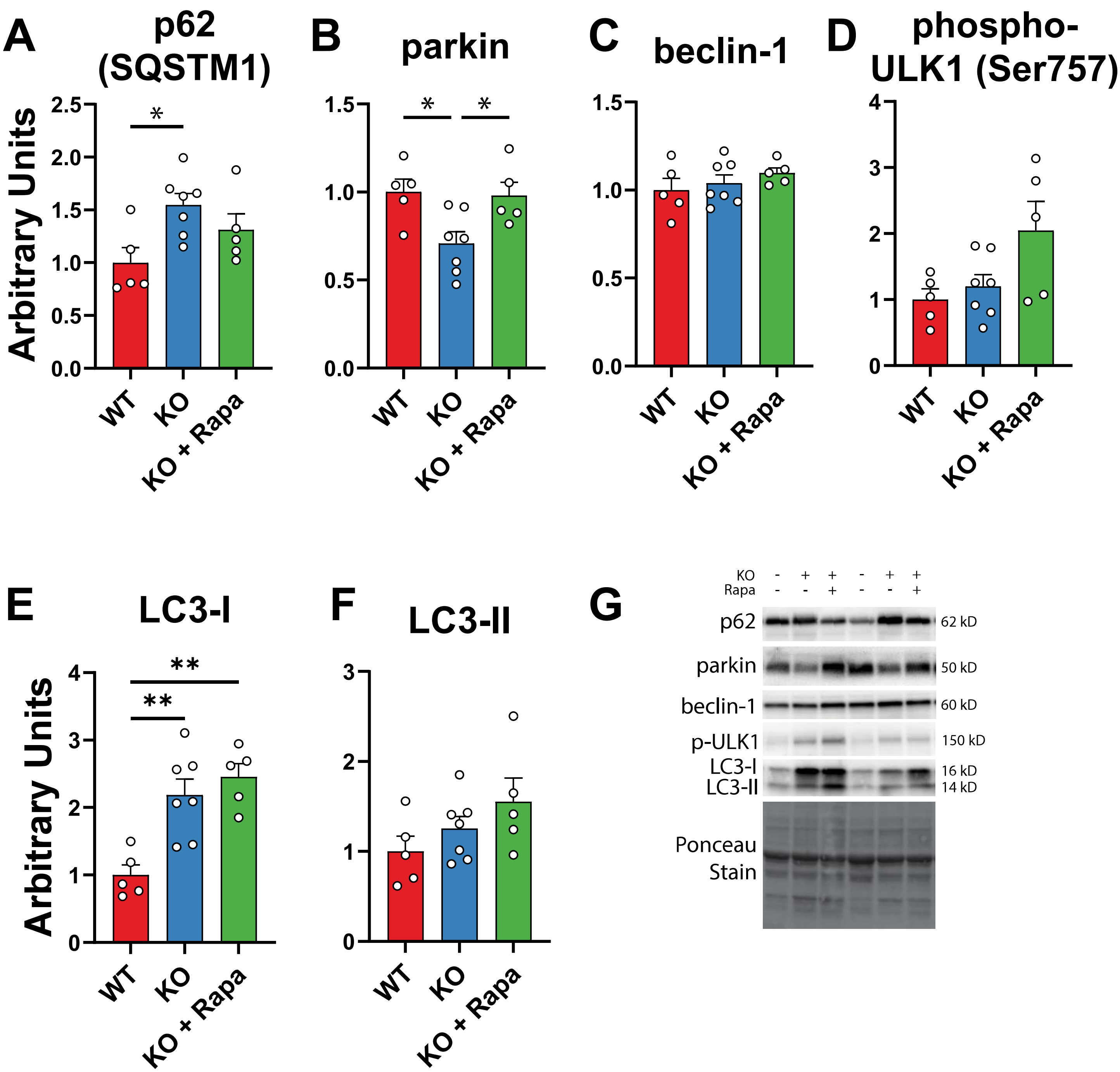

A
p62 (SQSTM1)
B
parkin
C
beclin-1
D
phospho-ULK1 (Ser757)
E
LC3-I
F
LC3-II
G
Arbitrary Units
WT
KO
KO + Rapa
KO
Rapa
p62
parkin
beclin-1
p-ULK1
LC3-I
LC3-II
Ponceau Stain
62 kD
50 kD
60 kD
150 kD
16 kD
14 kD

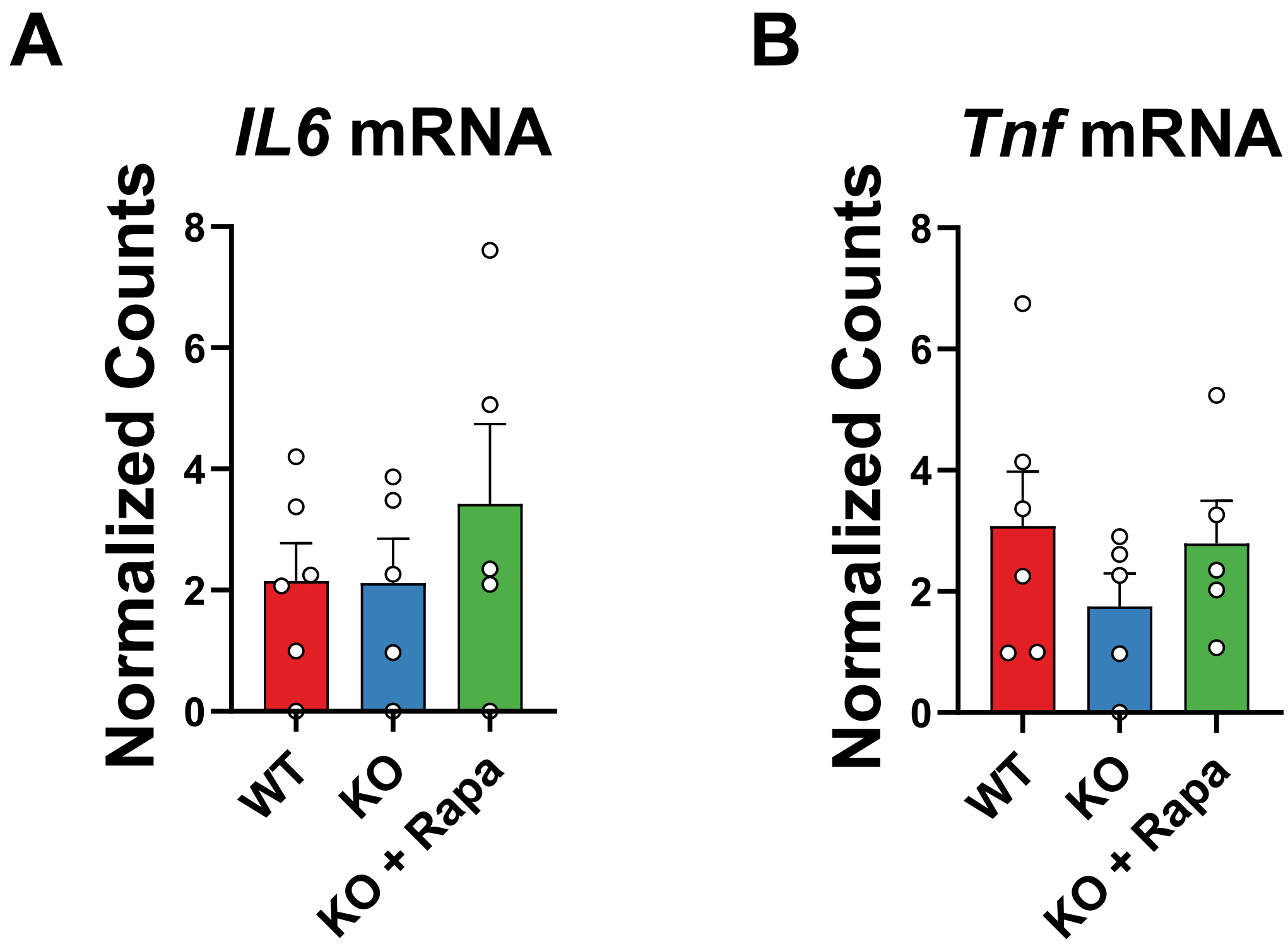
A
IL6 mRNA
Normalized Counts
8
6
4
2
0
WT
KO
KO + Rapa
B
Tnf mRNA
Normalized Counts
8
6
4
2
0
WT
KO
KO + Rapa

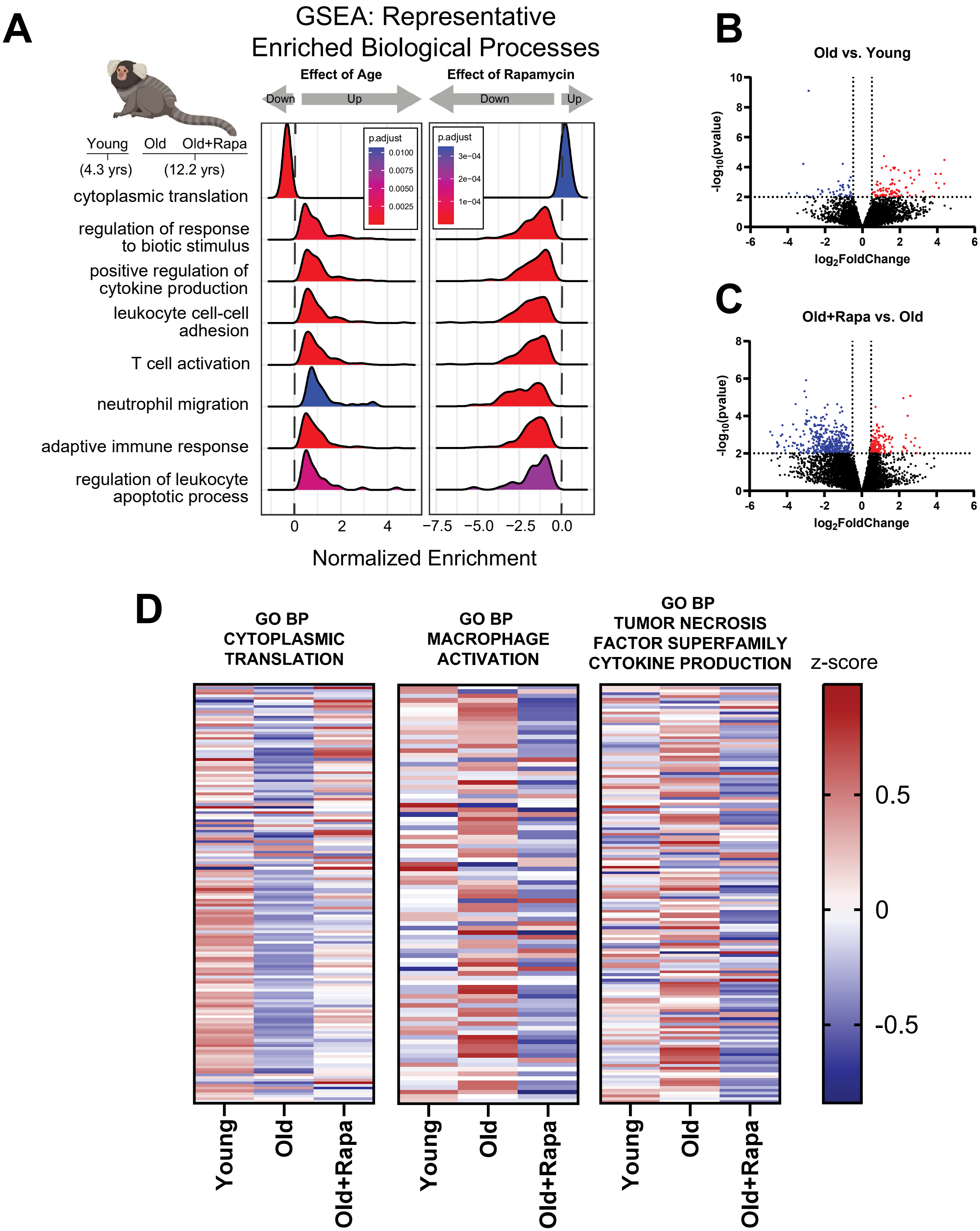
A
GSEA: Representative Enriched Biological Processes
Effect of Age
Down
Up
Effect of Rapamycin
Down
Up
Young
Old
Old+Rapa
(4.3 yrs)
(12.2 yrs)
cytoplasmic translation
regulation of response to biotic stimulus
positive regulation of cytokine production
leukocyte cell-cell adhesion
T cell activation
neutrophil migration
adaptive immune response
regulation of leukocyte apoptotic process
p.adjust
0.0100
0.0075
0.0050
0.0025
p.adjust
3e−04
2e−04
1e−04
0
2
4
−7.5
−5.0
−2.5
0.0
Normalized Enrichment
B
Old vs. Young
-log10(pvalue)
log2FoldChange
C
Old+Rapa vs. Old
-log10(pvalue)
log2FoldChange
D
GO BP CYTOPLASMIC TRANSLATION
GO BP MACROPHAGE ACTIVATION
GO BP TUMOR NECROSIS FACTOR SUPERFAMILY CYTOKINE PRODUCTION
z-score
0.5
0
-0.5
Young
Old
Old+Rapa
