# Supplementary material for: Constitutive Activation of Skeletal Muscle mTORC1 Drives Pro‐Inflammatory Gene Expression in Skeletal Muscle of Aged Mice
Source: Aging Cell. 2026 Nov 6;25(11):e70805. doi: 10.1111/acel.70805 (PMC13741394; doi:10.1111/acel.70805)

LC = Loading Control WT = Wild Type KO = Knockout Rapa = Rapamycin

Beclin Gel 1

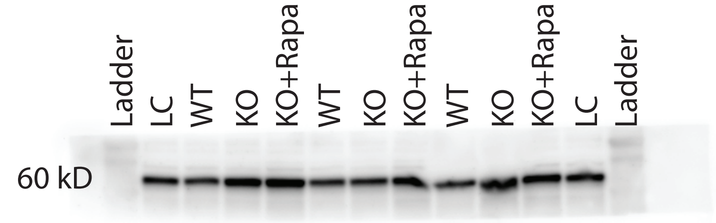

Beclin Gel 2

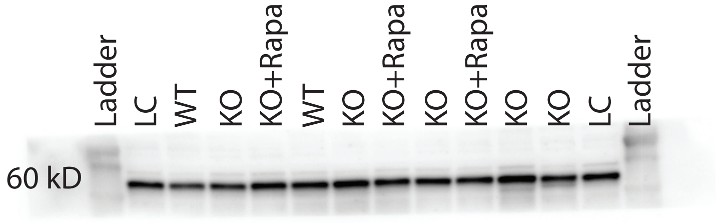

TNF-α Gel 1

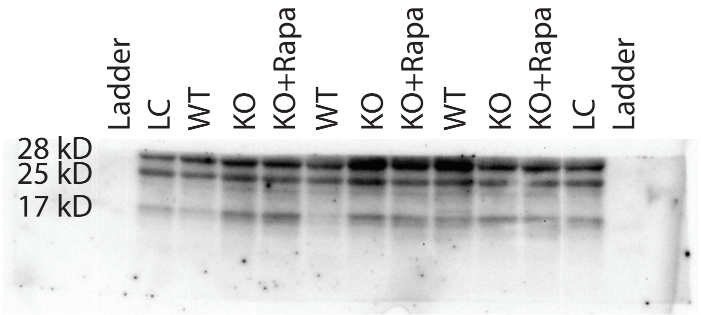

TNF-α Gel 2

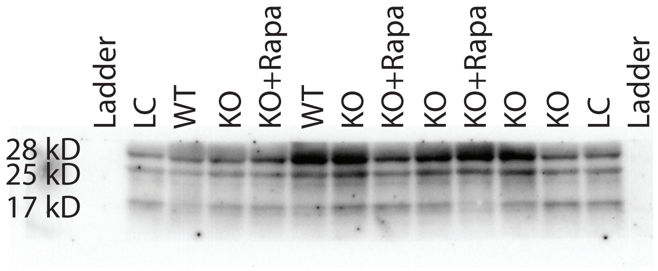

Ponceau Gel 1 (For Beclin and TNF-α)

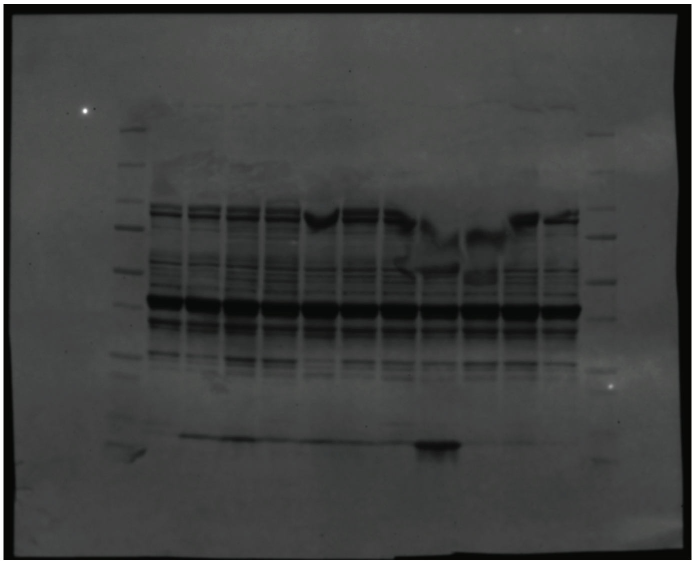

Ponceau Gel 2 (For Beclin and TNF-α)

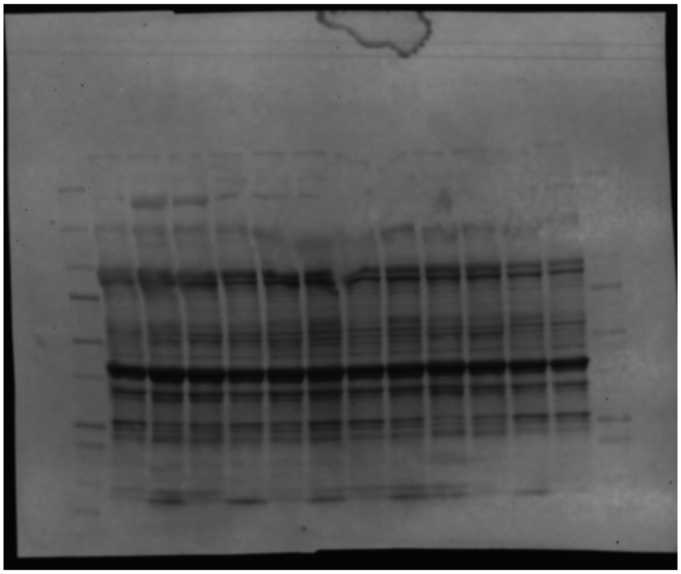

phospho-ULK1 Gel 1

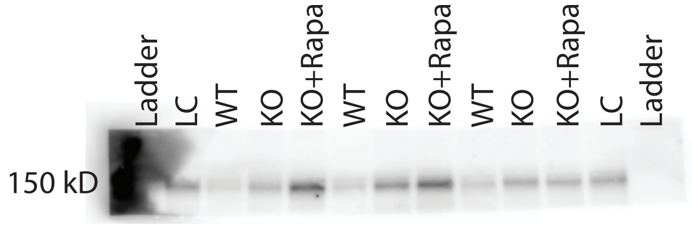

phospho-ULK1 Gel 2

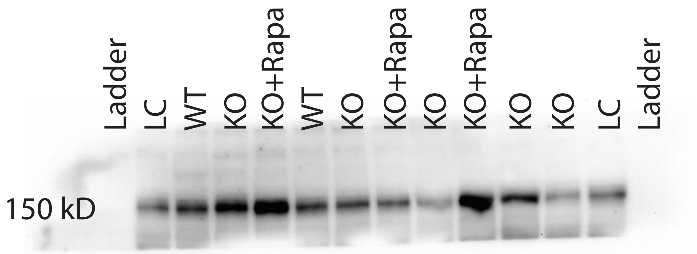

P62 Gel 1

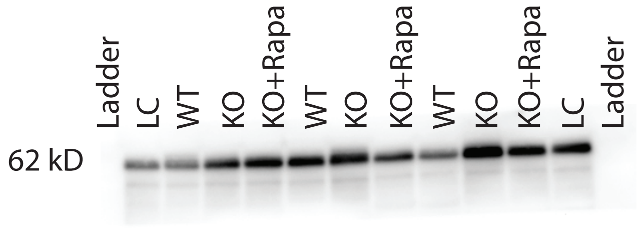

P62 Gel 2

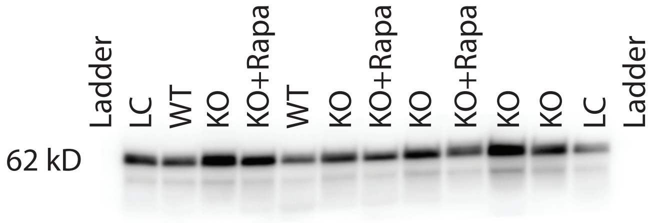

Ponceau Gel 1 (For P62 and phospho-ULK1)

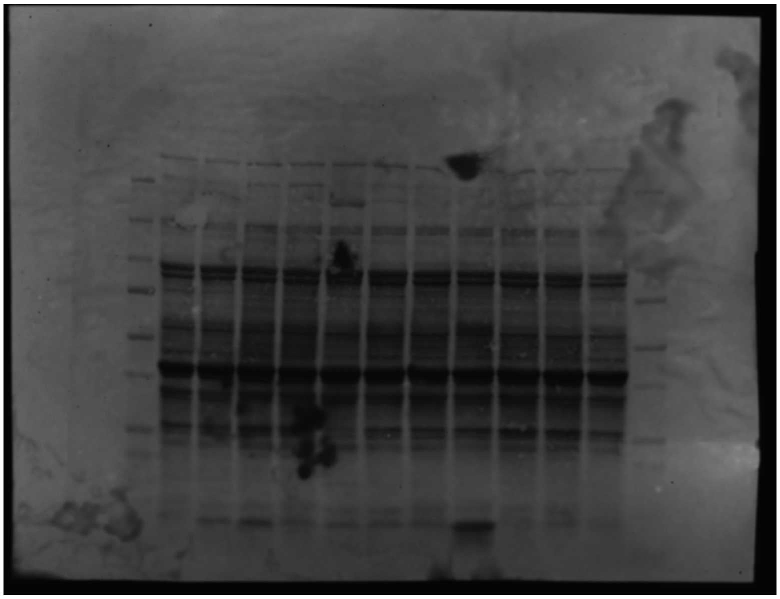

Ponceau Gel 2 (For P62 and phospho-ULK1)

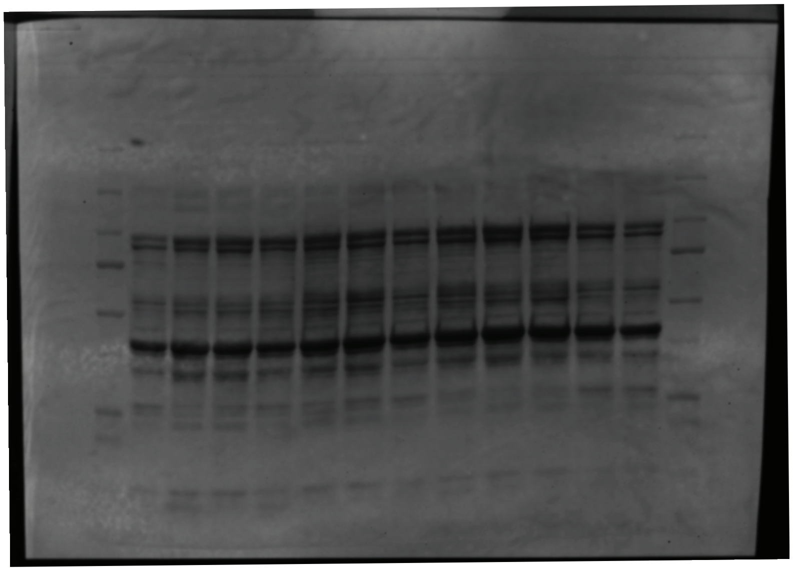

Parkin Gel 1

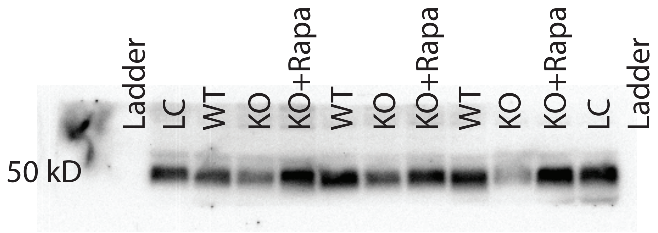

Parkin Gel 2

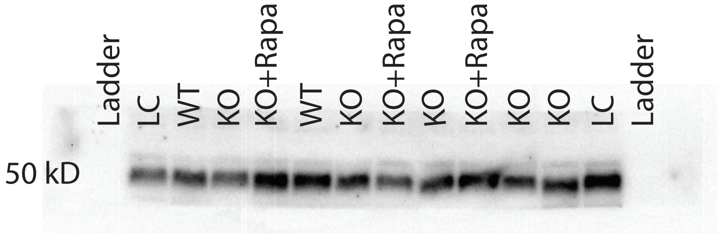

Ponceau Gel 1 (For Parkin)

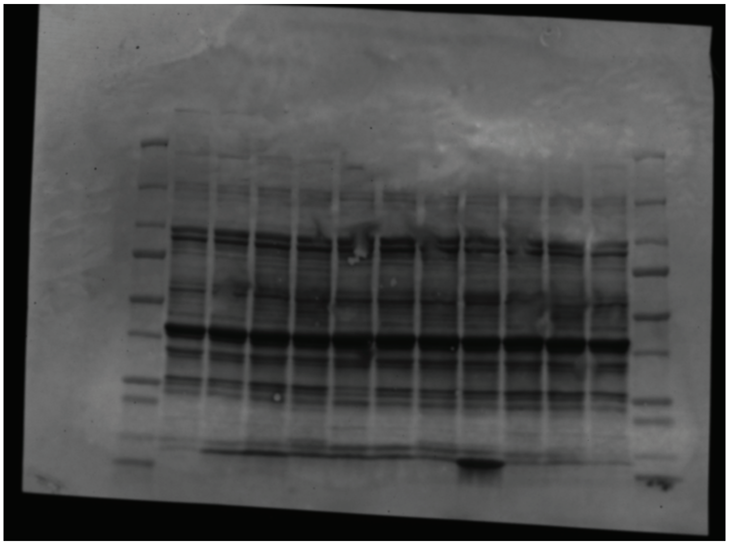

Ponceau Gel 2 (For Parkin)

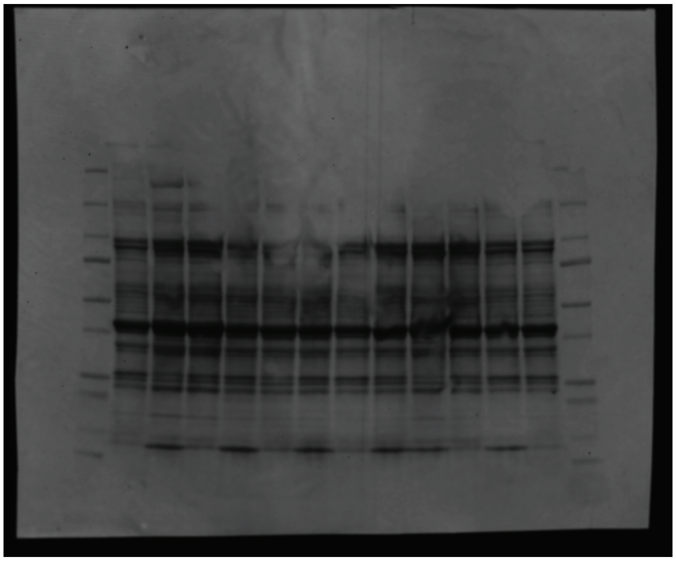

LC3 Gel 1

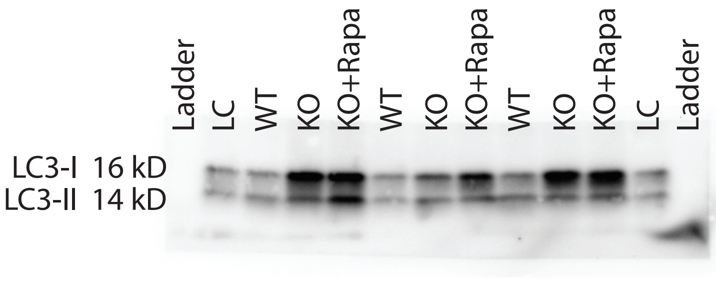

LC3 Gel 2

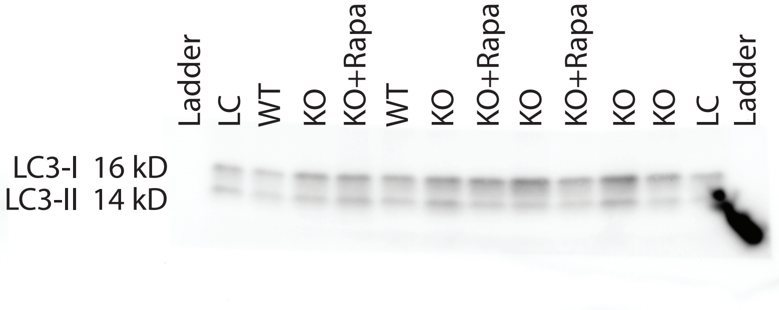

Ponceau Gel 1 (For LC3)

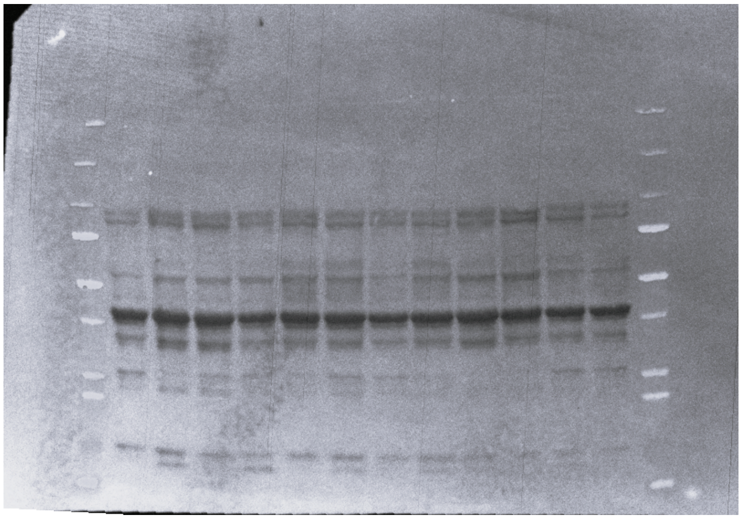

Ponceau Gel 2 (For LC3)

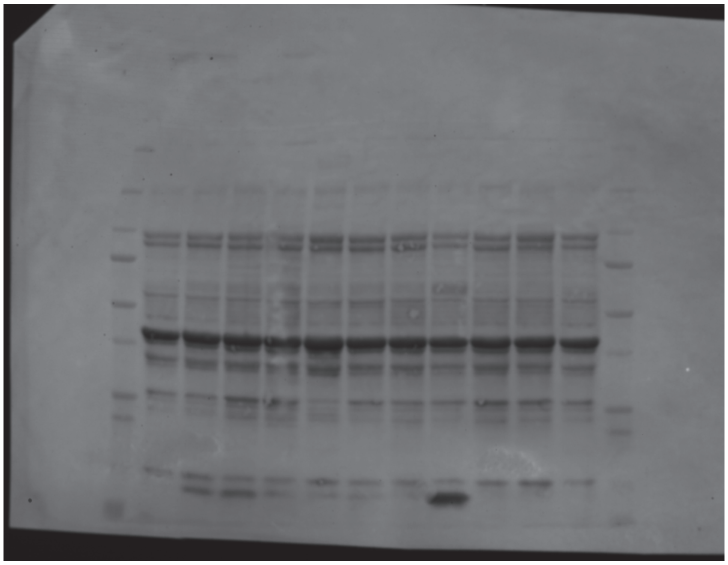

phospho-S6 Gel 1

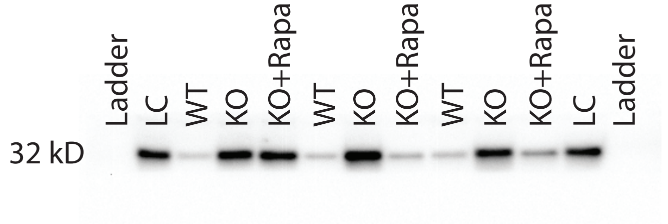

phospho-S6 Gel 2

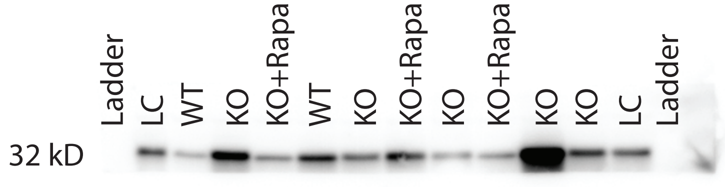

Total 4E-BP1 Gel 1

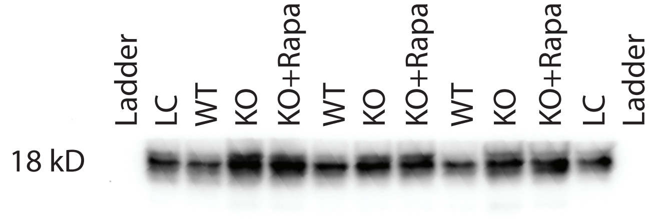

Total 4E-BP1 Gel 2

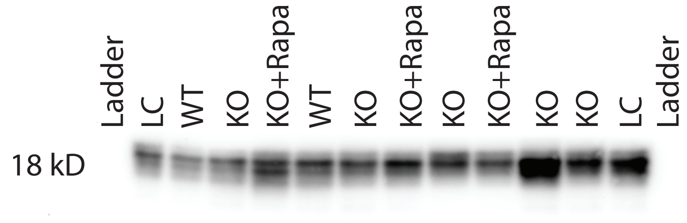

Ponceau Gel 1 (For phospho-S6 and Total 4E-BP1)

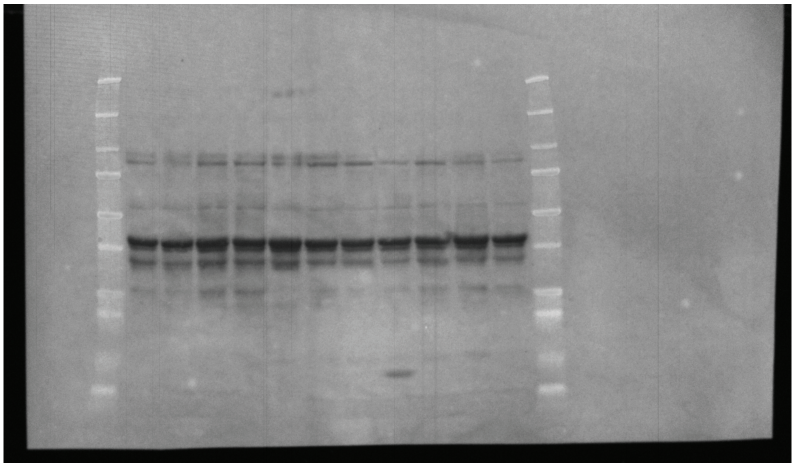

Ponceau Gel 2 (For phospho-S6 and Total 4E-BP1)

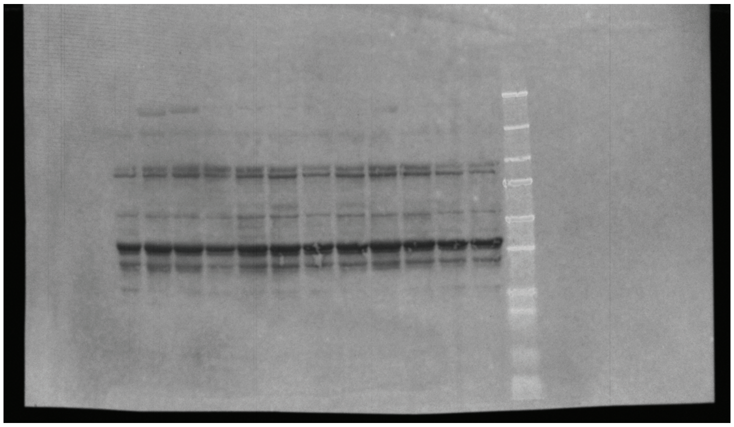

Total-S6 Gel 1

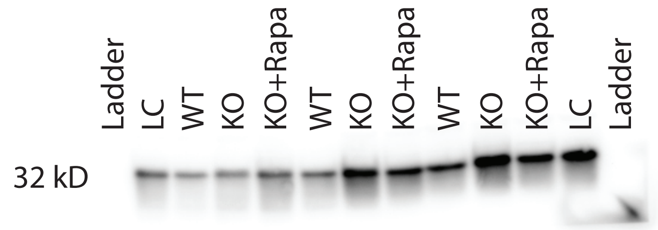

Total-S6 Gel 2

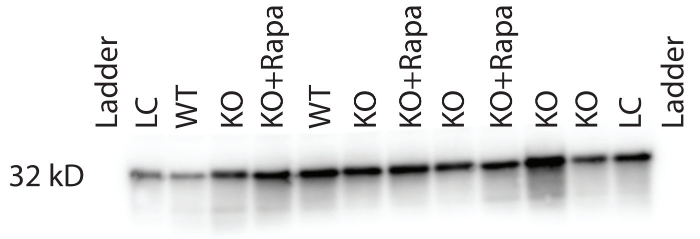

Phospho-4E-BP1 Gel 1

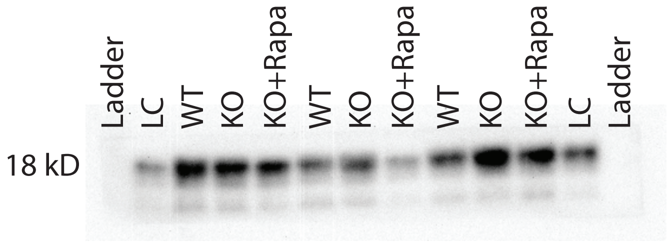

Phospho-4E-BP1 Gel 2

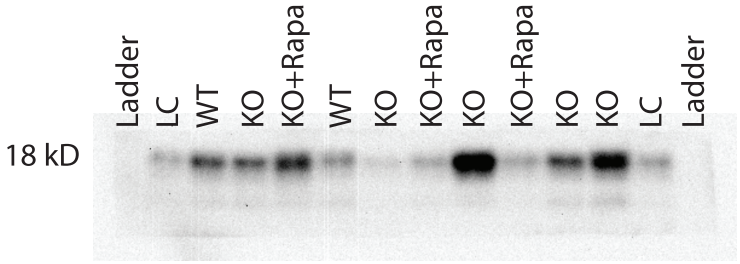

Ponceau Gel 1 (For Total-S6 and Phospho-4E-BP1)

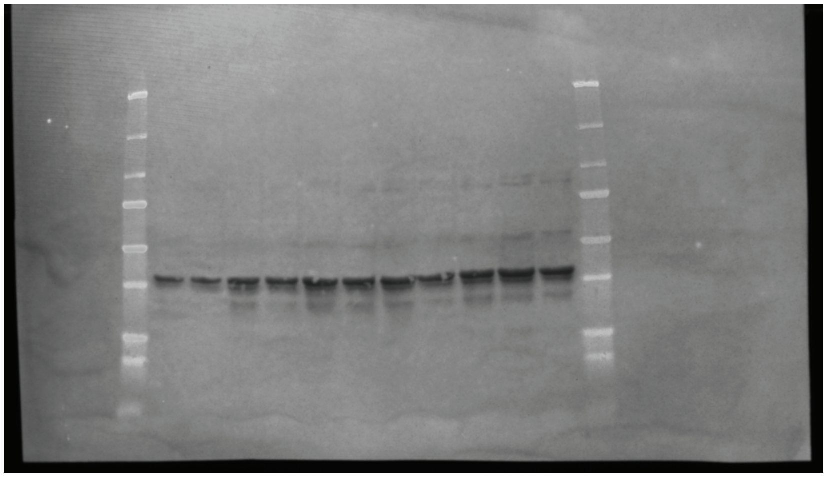

Ponceau Gel 2 (For Total-S6 and Phospho-4E-BP1)

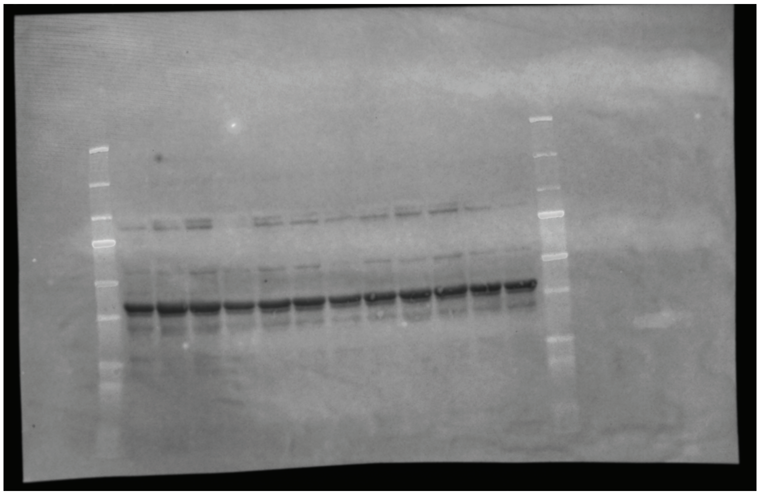

IL-6 Gel 1

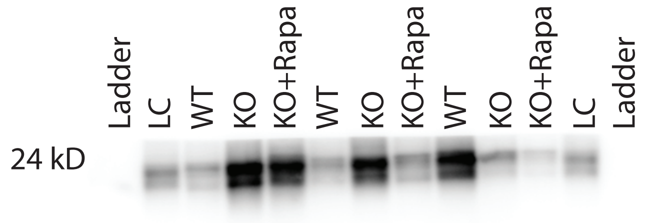

IL-6 Gel 2

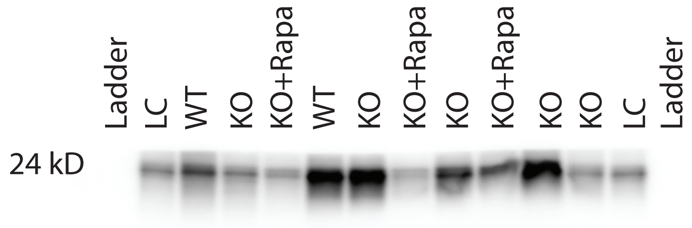

DEPDC5 Gel 1

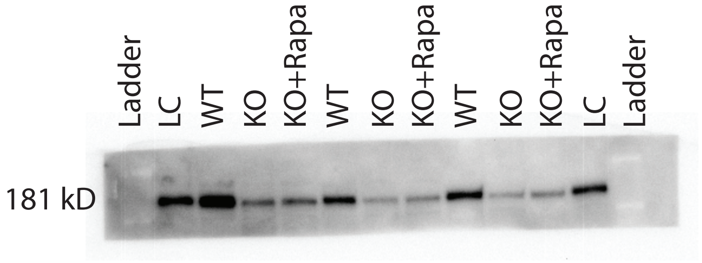

DEPDC5 Gel 2

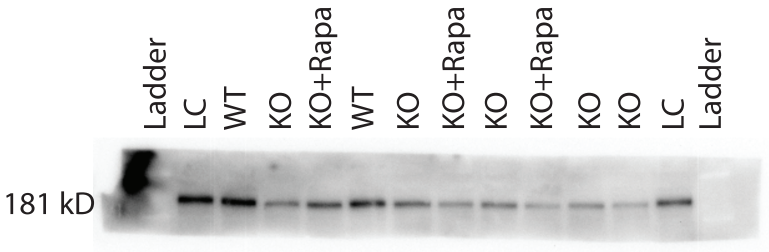

Ponceau Gel 1 (For IL-6 and DEPDC5)

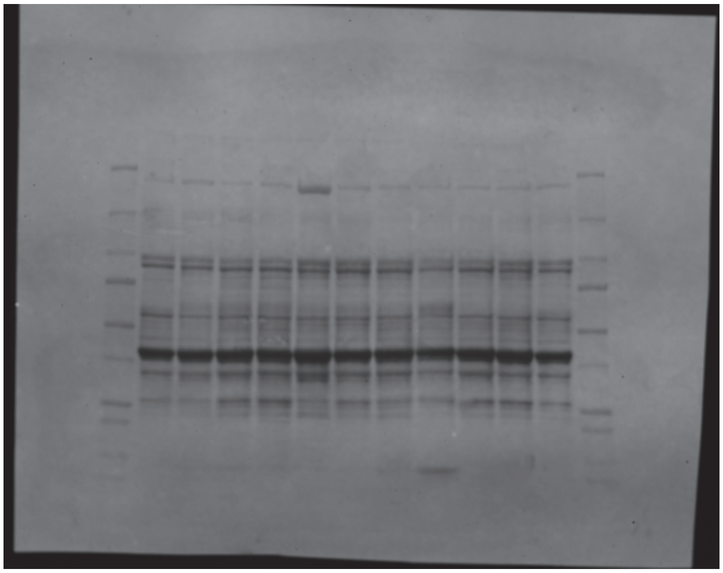

Ponceau Gel 2 (For IL-6 and DEPDC5)

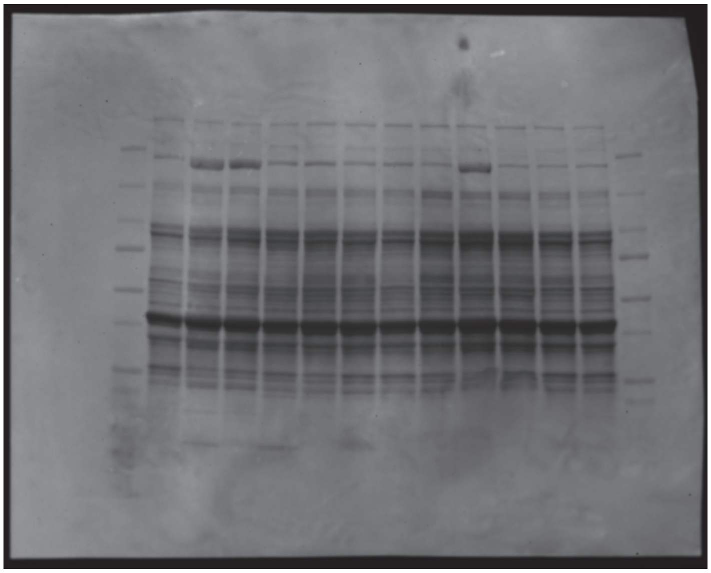

Supplement: Supplementary file 2 [file ACEL-25-e70805-s001.zip › Uncropped Western Blot Images.pdf]
